# Definition of Laser Acupuncture and All Kinds of Photo Acupuncture

**DOI:** 10.3390/medicines5040117

**Published:** 2018-10-30

**Authors:** Gerhard Litscher

**Affiliations:** Research Unit for Complementary and Integrative Laser Medicine, Research Unit of Biomedical Engineering in Anesthesia and Intensive Care Medicine, and TCM Research Center Graz, Medical University of Graz, Auenbruggerplatz 39, EG19, 8036 Graz, Austria; gerhard.litscher@medunigraz.at; Tel.: +43-316-385-83907; Fax: +43-316-385-595 83907

**Keywords:** laser acupuncture, photo acupuncture, definition, photobiomodulation

## Abstract

This editorial contains a general definition of laser acupuncture and all kinds of photo acupuncture accepted by the World Association for photobiomoduLation Therapy (WALT).

The number of studies on laser acupuncture listed in the SCI and PubMed databases is steadily increasing. Altogether, in PubMed, the most important medical database (www.pubmed.gov), there are 921 publications on this topic as of 20 October 2018. However, at the moment, there is no common accepted definition of the term laser acupuncture.

On 5 October 2018, the following general definition of laser acupuncture was developed and discussed during a consensus session (Chairman of the session: Prof. Gerhard Litscher) at the 12th International WALT (World Association for photobiomoduLation Therapy) Congress in Nice (Figure 1), in France. In this session, the chairman, all invited speakers, and, in addition, 28 experts from around the world agreed. The next day (6 October 2018), among other things, the proposal was presented by the chairman in the context of another consensus session and the entire executive board of WALT (Chair: Prof. Praveen Arany, President of WALT and Co-Chair of the Congress and Prof. Rene-Jean Bensadoun, Co-Chair of the Congress) also approved the proposal. Here is the definition:

Definition of Laser Acupuncture *:

**“Photonic stimulation of acupuncture points and areas to initiate therapeutic effects similar to that of needle acupuncture and related therapies together with the benefits of PhotoBioModulation (PBM)”**

*^)^ and all kinds of Photoacupuncture

The basics of laser acupuncture are well described in scientific literature [1,2,3,4]. In addition, it has also been shown that laser acupuncture and needle acupuncture in healthy participants are able to produce different brain patterns [5]. Laser acupuncture activates the precuneus relevant to mood in the posterior default mode network, while needle acupuncture activates the parietal cortical region associated with the primary motor cortex. Further investigations are warranted to evaluate the clinical relevance of these effects [5].

It is also appropriate to indicate here that WALT guidelines will be coming out shortly (Summer 2019) on the current state of knowledge with primarily human treatment studies specifically, but also evidence from lab studies.

## Figures and Tables

**Figure 1:**
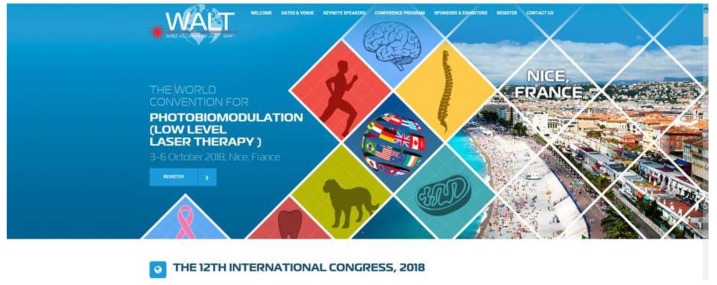
**W**orld **A**ssociation for photobiomodu**L**ation **T**herapy (W.A.L.T.). 12th International Congress, Nice, France, 3–6 October 2018 (© WALT).
